# TORC1, stress and the nucleolus

**DOI:** 10.18632/aging.101456

**Published:** 2018-05-22

**Authors:** Emiliano Matos-Perdomo, Félix Machín

**Affiliations:** 1 Unidad de Investigación, Hospital Universitario Ntra Sra de Candelaria, 38010 Santa Cruz de Tenerife, Spain; 2 Universidad de La Laguna, 38200 Tenerife, Spain

**Keywords:** TORC1, ribosomal DNA (rDNA), heat stress, stress responses

The nucleolus, a membrane-free organelle embedded inside the nucleus, has been classically considered as a mere ribosome factory; however, an emerging body of evidence points nowadays towards a more active role in cell homeostasis [1]. The nucleolus is organized around the nucleolar organizing regions (NORs), which are chromosomal regions of ribosomal DNA (rDNA) clustered and repeated in tandem. In the budding yeast *Saccharomyces cerevisiae*, the rDNA is located on the right arm of chromosome XII, the largest chromosome in this organism. Since transcription of the rDNA and extensive processing of the nascent rRNAs take place temporally and spatially in tight association, the rDNA and the nucleolus represent the same entity in budding yeast (as seen by fluorescence microscopy or Miller spreads). The rDNA can be visualized by FISH or by means of specific proteins that bind to the rDNA (e.g. Net1-GFP) and has been used in the past as a model for chromosome structure and condensation. Upon treatment with the microtubule depolymerizing agent nocodazole, which arrest cells in metaphase, the rDNA adopts a “loop” structure away from the nuclear mass stained with DAPI. Similar to some extent, the nuclear membrane adjacent to the nucleolus adopt a structure designated as the “nuclear flare”.

TOR, the central controller of metabolism in the cell is composed by two complexes, TORC1 and TORC2. TORC1 complex promotes multiple energy-expensive processes require for cell growth, including rDNA transcription and ribosome biogenesis, while inhibiting others like autophagy and stress responses. This complex integrates nutritional and environmental inputs in the cell, thus being affected by nutritional status, amino acids, abiotic stress, etc.

Some authors claim that the nucleolus could act as a general sensor of stress and, in addition, an rDNA theory of aging has been proposed [2]. This theory includes that TOR inhibition (rapamycin treatment) and dietary restriction have an impact on the nucleolar and rDNA structure [3]. The proposed mechanism behind this is that the stabilization of the rDNA upon TOR inhibition and/or calorie restriction (CR) leads to lifespan extension. Moreover, both CR and TOR turned out to be part of the same longevity pathway by increasing sirtuins activity (Sir2/Hst2) [4].

An intriguing phenotypic feature of CR and rapamycin treatment was the compaction seen on the nucleolus/rDNA both in cycling or mitotic arrested cells [5,6]. How and why would a common pathway control the structure and stability of the rDNA, and if so, be extended to other kind of stresses? We shed light on this in a recent work, extending also the spectrum of stresses that could have an effect on the rDNA [6]. Remarkably, heat stress (HS) was not consider of importance when making use of temperature conditional alleles, especially regarding to the structure of the rDNA. However, surprisingly, the same phenotypic effect on the rDNA seen by rapamycin and CR is observed upon acute HS. Other stresses known to inhibit TOR signalling like nitrogen starvation, carbon starvation and oxidative stress have similar effects.

A different scenario was seen for the protein folding stressor AZC (L-Azetidine-2-carboxylic acid), a proline analogue. The treatment with AZC did not cause the compaction phenotype seen with other stresses. A recent work demonstrates that misfolded proteins trigger TORC1 active, acting as a messenger of the folding environment, whereas improved proteostasis renders TORC1 inactive, acting in this case as a sensor of the improvement in the folding environment [7]; similar conclusions were obtained in mammals in another study. Since proteostasis mainly depends on Heat Shock Factor 1 (HSF1), it would be interesting to ascertain the role of HSF1 on rDNA structure/stability and the interplay with TORC1.

**Figure 1:**
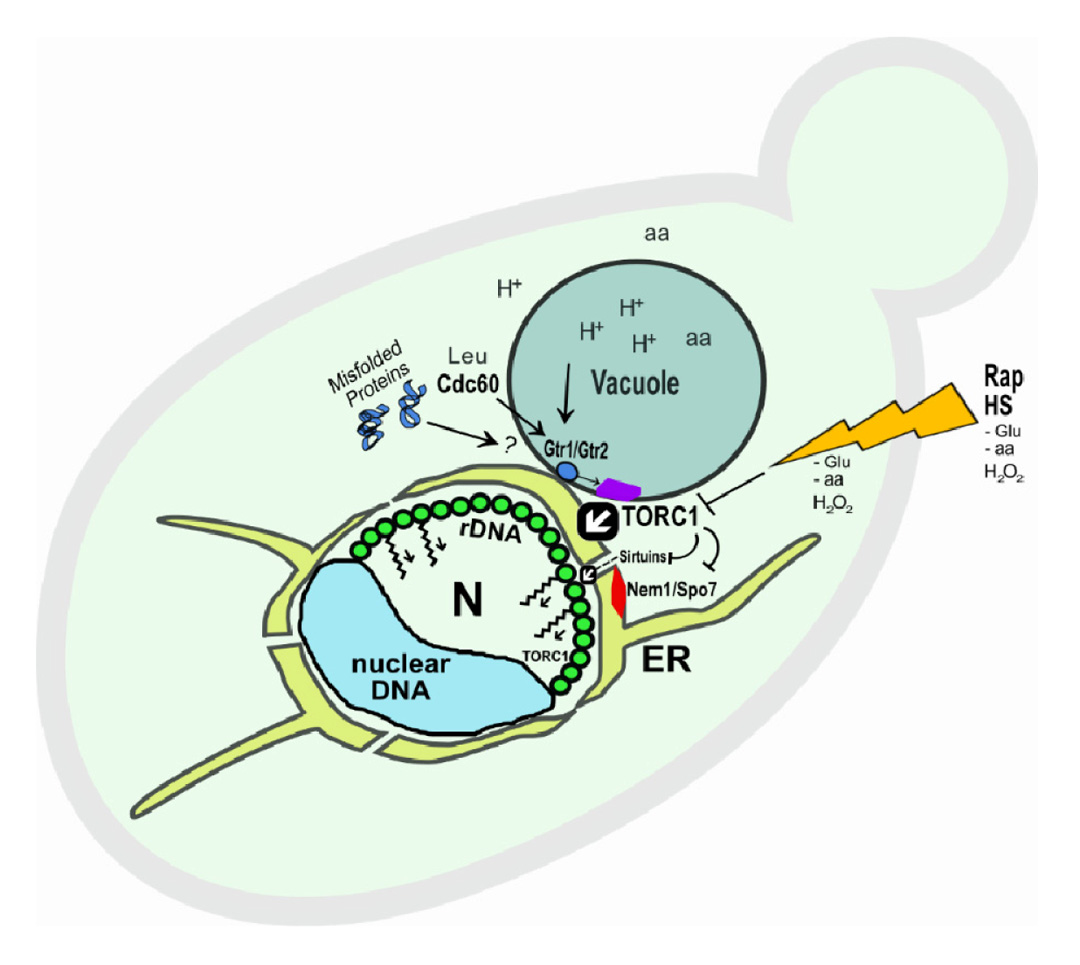
**Hypothetical model of TORC1 mediated control on several processes affecting the rDNA structure.** A metaphase arrest in yeast yields a characteristic rDNA loop (green knobs). TORC1 stimulates transcription of rDNA. Although TORC1 complex is mainly located at the vacuole/lysosome (purple rectangle), pools could also exist in the nucleus (N) including at the rDNA. Stimulated TORC1 would impinge on the rDNA structure, while blocking sirtuins and Nem1/Spo7-Pah1 complex. Upon rapamycin treatment or calorie restriction, or by means of stresses like Heat Stress (HS), the rDNA shrinks into a compacted structure (not shown). TORC1 inhibition would lead to an increasing activity of sirtuins, and the Nuclear/Endoplasmic reticulum (ER) membrane would shrink as well, by altering Nem1-mediated lipid deposition. This latter effect would be apparently restricted to the rDNA/nucleolar region. The kinked lines with arrows would indicate compaction of the rDNA under TORC1 inhibition. White arrows within black boxes indicate specific actions on the rDNA.

In the case of amino acid limitation, one amino acid (Leucine) has been linked to TORC1 activation, through the Leucyl tRNA synthetase Cdc60, and to autophagy inhibition. Importantly, we showed that Cdc60 auxin-mediated depletion directly caused rDNA compaction without an external stress. Cdc60 acts via the EGO complex components Gtr1/2, which are also stimulated by vacuolar proton V-ATPases. Increasing evidence puts TORC1/Sch9 and the vacuole in a circuit coupling pH homeostasis and glucose/nutrient availability. Thus, it could be possible that protein synthesis, pH homeostasis and nutrient availability could be controlled by a few core components. The nuclear flare extension can be seen on certain mutants; i.e., for the Nem1/Spo7 complex and the Pah1 phosphatase involved in Nuclear/ER membrane expansion, phospholipids synthesis, lipid droplet formation, triacylglycerol synthesis, etc. Interestingly, Nem1/Spo7 is under the control of TORC1. Therefore, protein biosynthesis and lipid biogenesis could be regulated in a concerted manner with the aforementioned processes (Figure 1).

In conclusion, the rDNA gets compacted upon stresses that inactivate TORC1. A range of TORC1 coordinated/sensed processes impinge on the nucleolar/rDNA structure, probably linking those processes to the stability of the rDNA, increased stress response and lifespan extension.
